# Modulatory Effects of Regulated Cell Death: An Innovative Preventive Approach for the Control of Mastitis

**DOI:** 10.3390/cells13201699

**Published:** 2024-10-14

**Authors:** Xiaojing Xia, Pengfei Ren, Yilin Bai, Jingjing Li, Huihui Zhang, Lei Wang, Jianhe Hu, Xinwei Li, Ke Ding

**Affiliations:** 1 Henan Institute of Science and Technology, College of Animal Science and Veterinary Medicine, Xinxiang 453003, China; quik500@163.com (X.X.);; 2 Laboratory of Indigenous Cattle Germplasm Innovation, School of Agricultural Sciences, Zhengzhou University, Zhengzhou 450001, China; 3 College of Veterinary Medicine, Jilin University, Changchun 130062, China

**Keywords:** mastitis, regulated cell death, apoptosis, autophagy, pyroptosis, ferroptosis, necroptosis

## Abstract

Mastitis is a common disease worldwide that affects the development of the dairy industry due to its high incidence and complex etiology. Precise regulation of cell death and survival plays a critical role in maintaining internal homeostasis, organ development, and immune function in organisms, and regulatory abnormalities are a common mechanism of various pathological changes. Recent research has shown that regulated cell death (RCD) plays a crucial role in mastitis. The development of drugs to treat cell death and survival abnormalities that can be widely used in mastitis treatment has important clinical significance. This paper will review the molecular mechanisms of apoptosis, autophagy, pyroptosis, ferroptosis, and necroptosis and their regulatory roles in mastitis to provide a new perspective for the targeted treatment of mastitis.

## 1. Introduction

Mastitis is a common disease in dairy farms. After the mammary gland is infected with pathogenic microorganisms or stimulated by physical, chemical, and other factors, breast interstitial or parenchymal tissue undergoes inflammatory changes [1]. Sick cows will be eliminated due to decreased milk production or loss of lactation ability. Mastitis seriously affects the milk yield of dairy cows and the quality of dairy products and has long plagued the dairy industry [2,3]. The etiology of mastitis is complex, and pathogenic microbial infection is the main cause of this disease. More than 80% of mammary gland infections are caused by bacteria [1,4,5]. In addition, mastitis induced by viruses, mycoplasma, fungi, and other infections has been reported [1,4,5]. Conventional treatment methods include physical therapy, traditional Chinese medicine, and antibiotic therapy. The scope of physical therapy is relatively limited and is not suitable for hemorrhagic and suppurative mastitis. Traditional Chinese medicine has almost no residue, does not pollute the environment, does not lead to resistance, and has few side effects. However, the curative effect of Chinese medicine treatment is slow, and the treatment period is long and requires a large dose [6]. Antibiotics are the first-choice treatment and the best way to treat mastitis because of the high cure rate and excellent curative effect. However, the long-term, widespread use of antibiotics can lead to a series of side effects, such as antibiotic residues, increased resistance to pathogenic microorganisms, and decreased milk quality, which pose significant safety risks to human health [6]. Therefore, it is urgent to seek safe and effective treatments for mastitis.

**Figure 1:**
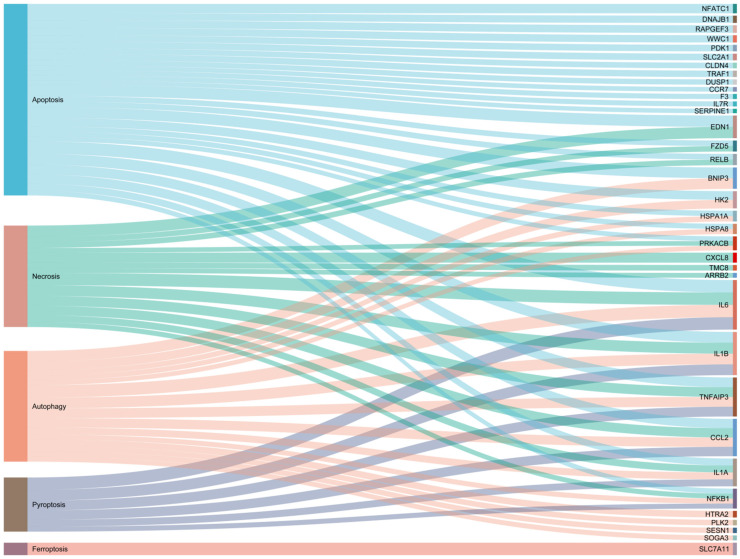
The cell death-related gene list contains 36 genes, of which 27 are from apoptosis, 14 are from necroptosis, 6 are from pyroptosis, 15 are from autophagy, and 1 is from ferroptosis.

Normal cell death is necessary to maintain the normal physiological function of the organism, while abnormal cell death leads to an excessive inflammatory response and dysfunction in the organism [7,8]. Regulated cell death (RCD), including autophagy, apoptosis, ferroptosis, pyroptosis, necroptosis, and panoptosis, is widely involved in the normal physiological activities of the body and is closely correlated with the occurrence and development of many diseases [7,8,9]. Studies have shown that the occurrence and development of mastitis are accompanied by various RCDs involving signaling pathway activation, the release of inflammatory factors, and the self-regulation and repair of the organism. For example, the invasion of pathogenic bacteria can induce apoptosis in a large number of BMECs, causing mastitis [10]. Neutrophil apoptosis is beneficial for alleviating inflammation, but if apoptosis is delayed by the influence of pathogenic microorganisms, acute mastitis will develop into chronic or subclinical mastitis [11]. Autophagy can ameliorate inflammatory damage in organisms by maintaining organelle stability [12]. During mastitis, pyroptosis expands inflammation, and excessive pyroptosis can cause multiple organ failure [13]. The antioxidant capacity of cells decreases, and lipid reactive oxygen species (ROS) can cause iron-dependent oxidative cell death [14]. Necroptosis also exists in breast tissue, in which mastitis occurs [15]. Based on the transcriptome sequencing (RNA-Seq) technique, our research team found 36 genes related to cell death were found in the study of lipopolysaccharide (LPS)-induced inflammatory response of bovine mammary epithelial cells (BMECs), among which 27 were associated with apoptosis, 14 with necroptosis, 6 with pyroptosis, 15 with autophagy, and 1 with ferroptosis (Figure 1). These studies suggest that there is an important association between RCD and mastitis. The process of cell death is regulated by various signaling pathways, and RCD can be treated through pharmacological or genetic approaches, which has become a research hotspot in this field. Combined with the latest research progress, this article discusses the relationship between apoptosis, autophagy, programmed necroptosis, pyroptosis, ferroptosis, and mastitis, as well as interventions with related drugs, in an effort to provide new perspectives for the prevention and treatment of mastitis.

## 2. Apoptosis

Apoptosis is the most intensively studied RCD, and cells die actively under gene regulation with clear characteristics [16]. In the early stage, cell surface microvilli disappear, membrane protrusions vacuolate, the cytoplasm becomes concentrated, cell chromatin condenses, the plasma membrane roughens, and the cell gradually shrinks in size and detaches from the surrounding cells [17]. In the late stage, internuclear DNA breaks, the cell membrane is depressed, and nuclear fragments and organelles are wrapped to form apoptotic bodies with complete membrane components, which can be quickly identified and phagocytized by adjacent phagocytes [18]. The formation of apoptotic bodies is an important feature of apoptosis [19].

### 2.1. Overview of the Core Mechanisms of Apoptosis

There are three major apoptotic signal transduction pathways: the endoplasmic reticulum pathway, the mitochondrial pathway, and the death receptor pathway. In the death receptor pathway, caspase-8 is activated through the death receptor on the cell membrane and then activates the downstream effector caspase-3 to induce apoptosis. In the mitochondrial pathway, cytochrome C (Cytc) is released from mitochondria, and then cytochrome c forms a complex with an apoptotic protease activator, and the precursor of caspase-9 is recruited. After the activation of caspase-9, downstream caspase-3 is activated and induces apoptosis. After the endoplasmic reticulum calcium balance is destroyed, the endoplasmic reticulum pathway is activated. Subsequently, caspase-12 expression is upregulated, and caspase-7 is transferred to the surface of the endoplasmic reticulum, where it leads to caspase-12 cascade activation, which further leads to caspase-3 activation and induces apoptosis [20]. The three apoptosis signal transduction pathways all activate the downstream effector caspase-3 through a chain of signal transduction, ultimately initiating apoptosis [21]. In addition, apoptosis is tightly regulated by the Bcl-2 protein family, which is composed of members with proapoptotic or antiapoptotic functions, such as the proapoptotic proteins bax, bad, and bcl-xs and the antiapoptotic proteins bcl-2, bxl-xl, and mcl-1 [11].

### 2.2. Apoptosis in Mastitis

Infection with pathogenic microorganisms is the main cause of mastitis. After pathogenic bacteria invade the body, the immune system will produce a series of immune reactions to eliminate pathogenic bacteria and reduce inflammation. During the process of pathogen infection, immune cells interact with pathogenic microorganisms, and apoptosis plays an important role [1,5]. On the one hand, the immune cells eliminate pathogenic microorganisms or limit further growth and reproduction through active apoptosis [17]. On the other hand, pathogenic microorganisms can induce apoptosis in immune cells involved in phagocytosis and sterilization to effectively avoid the attack of immune cells, maintain their own survival, and even expand the scope of infection [16,22]. Some pathogenic bacteria can accelerate apoptosis, such as *Escherichia coli* (*E. coli*), which can induce accelerated neutrophil apoptosis [16]. Extended-spectrum beta-lactamase-producing *E. coli* (ESBLEC) infects BMECs and induces apoptosis by upregulating intracellular reactive oxygen species (ROS) levels, decreasing mitochondrial membrane potential (MMP) expression, and upregulating the bax/bcl-2 ratio, which leads to BMEC injury [23]. Some bacteria slow the rate of apoptosis. For example, *Staphylococcus aureus* (*S. aureus*) uses its ability to survive neutrophil phagocytosis and delays neutrophil apoptosis by inhibiting the fusion of lysosomes and phagosomes in neutrophils. When neutrophils die, the release of cellular contents causes inflammatory reactions in surrounding cells, which leads to a second wave of attack on surrounding tissues. Sladeka et al. found that during mastitis caused by *S. aureus* infection, the number of apoptotic neutrophils and MPO^−^ macrophages increased significantly, but the mammary gland continued to be infected, which turned acute into chronic or subclinical symptoms [17]. Staphylococcal enterotoxin M amplifies the inflammatory response by upregulating the release of the proinflammatory cytokines IL-6 and tumor necrosis factor (TNF)-α, adhesion molecule ICAM-1, and chemokine MCP-1, and a sustained inflammatory response induces epithelial dysfunction [24].

During the development of mastitis, the apoptosis of some cells can reduce the inflammatory response and play a protective role, while the apoptosis of other cells will cause the inflammatory site to lose protection and be damaged. During mastitis, a large number of neutrophils migrate from the bloodstream to the mammary glands, and the neutrophil granules released at the infected site can damage and kill invading bacteria. However, neutrophils also undergo necrolysis and release cytotoxic particles into breast tissue to amplify the inflammatory response, which may lead to serious tissue damage [16]. In addition, the apoptosis of neutrophils is accompanied by the loss of some basic functions, such as reduced responsiveness to stimulation, weakened phagocytosis (including complete inability to swallow), decreased degranulation, and respiratory burst [17]. These basic functions are essential for maintaining the host’s defenses and responding to the damage caused by infectious pathogens. If neutrophil apoptosis is too rapid, the ability of the mammary immune system to fight infection may be weakened, and chronic infection may occur. The blood–milk barrier formed by BMECs is important for mammary gland resistance to bacterial infection. Apoptosis in BMECs leads to a decrease in barrier integrity, and destruction of the blood–milk barrier aggravates the development of mastitis [24].

### 2.3. Potential Therapeutic Applications of Apoptosis in Mastitis

**Figure 2:**
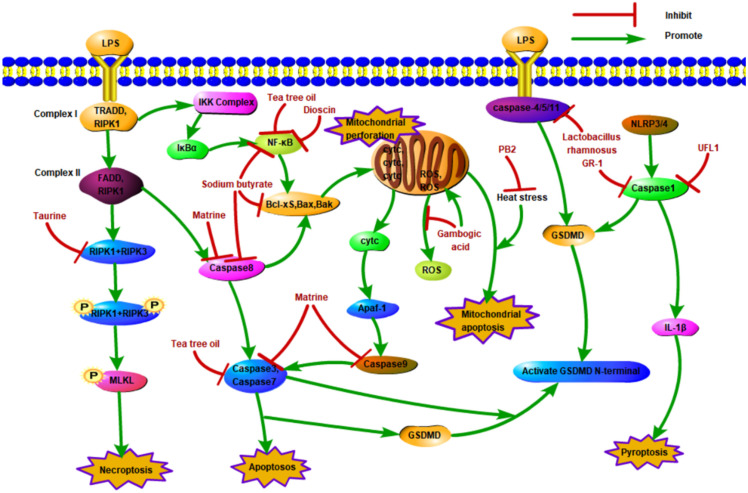
Therapeutic targets of core apoptosis, pyroptosis, and necroptosis proteins.

According to the molecular mechanism of apoptosis and the relationship between apoptosis and mastitis, researchers have tried to explore the treatment of mastitis by targeting apoptosis (Figure 2, Table 1). Gambogic acid, the main active ingredient of Garcinia cambogia, is known for its anticancer, anti-inflammatory, antibacterial, and neuromodulatory effects. Tang et al. showed that gambogic acid maintains stable mitochondrial membrane potential, inhibits excessive production of ROS, reduces apoptosis, and protects the blood–milk barrier from LPS-induced damage [25]. Matrine and baicalin are widely used in the clinic because of their anti-inflammatory, bacteriostatic, sensitizing, and immunological effects. It was found that matrine downregulated the expression levels of exogenous and endogenous cleaved caspase-9, cleaved caspase-8, and cleaved caspase-3, significantly inhibiting the apoptosis of BMECs induced by *S. aureus* or Panton–Valentine leukocidin (PVL) toxin. Baicalin can also reduce the apoptosis of BMECs by downregulating cleaved caspase-9 [20]. Chen et al. showed that tea tree oil downregulated the expression of caspase-3, mitogen-activated protein kinase 4 (MAPK4), and nuclear factor-κB in vitro, reduced apoptosis, and protected against the LPS-induced inflammatory response [26]. Bone marrow mesenchymal stem cells (MSCs) can be home to the injured site, play anti-inflammatory and anti-injury roles, and serve as carriers for gene therapy. Angiotensin-converting enzyme 2 (ACE2) plays a crucial role in a variety of inflammatory diseases and has strong anti-injury and anti-inflammatory effects. Yan et al. studied the anti-inflammatory effect of ACE2-modified MSCs on LPS-induced injury of BMECs. The results showed that MSC-ACE2 decreased the level of apoptosis-related proteins in EpH4-Ev cells and promoted repair of the blood–milk barrier after EPH4-EV cell injury [27]. In addition, MSCs-ACE2 have been shown to inhibit apoptosis by triggering the transcription 3/suppressor of cytokine signaling 3 (IL-10/STAT3/SOCS3) signaling pathway and reduce inflammatory damage to BMECs caused by *S. uberis* [21]. Sodium butyrate (SB), a short-chain fatty acid, has been shown to have anti-apoptotic, anti-inflammatory, and antioxidant effects on diverse cells in recent years. Ali et al. showed that sodium butyrate reduced LPS-induced apoptosis and inflammation by inhibiting the NF-kB and caspase/bax signaling pathways, suggesting that sodium butyrate can be used as a therapeutic agent for mastitis [28]. Proanthocyanidin B2 (PB2) is a phenolic compound with strong anti-inflammatory and antioxidant properties. Wang et al. found that PB2 could block mitochondrial apoptosis induced by heat stress (HS) and had an important protective effect on the HS-induced inflammatory response [29].

## 3. Autophagy

Autophagy, which is also known as type II programmed cell death (PCD), is a process in which cells undergo lysosomal-dependent degradation of cytoplasmic proteins, damaged organelles, excess lipids, and invasive pathogenic microorganisms, which are encapsulated by autophagosomes [40,41]. Lysosomes are monolayer-coated vesicles containing a variety of acid-hydrolytic enzymes that can degrade substances in autophagic lysosomes [42]. Autophagy is greatly conserved in eukaryotic cells and is regulated by autophagy-associated genes (ATGs), which play a critical role in cell growth, development, and death [43]. When cells are stimulated by hunger, high temperature, low oxygen, hormones, and other external stimuli or organelle damage, the accumulation of mutant proteins and invasion of microorganisms occurs, autophagy can be induced, and the contents of cells are digested to restore nutrients to cope with hunger. Studies have also shown that autophagy, which is a lysosome-dependent protein degradation pathway, can decrease inflammation by reducing inflammasome activation and inhibiting the level of proinflammatory cytokines [31].

### 3.1. Overview of the Core Mechanisms of Autophagy

Autophagy can be classified into chaperone-mediated autophagy (CMA), microautophagy, and macroautophagy according to different transport modes of intracellular autophagy substrates, and macroautophagy is the most widespread [44]. Under the modulation of ATGs and Beclin-1, autophagic vacuoles with double membrane structures engulf and package intracellular substances to form autophagosomes through the mediation of regulatory proteins such as P62. Microtubule-associated protein light chain 3 (LC3) is a specific molecular marker for autophagy. When autophagy is activated, LC3 is hydrolyzed by proteases and converted into the cytoplasmic protein of microtubule-associated protein light chain 3-I (LC3-I). The autophagy-related protein LC3-I binds with phosphatidylethanolamine to form lipidated LC3-II, which is then transferred to the autophagosome membrane. Subsequently, the autophagosome fuses with the lysosome to form an autophagolysosome with a monolayer membrane structure, which contains hydrolytic enzymes that further degrade the ingested contents [45]. Currently, research has found that over 30 autophagy-related genes (ATG) and proteins (Atg) are involved in the regulation of autophagy. LC3II protein activity is positively correlated with cellular autophagy levels and is a recognized marker reflecting autophagy activity [46]. Beclin1 is a homolog of yeast ATG6 and a core participant in autophagy regulation. During the formation, extension, and maturation of autophagosomes, Beclin1 forms a complex with phosphatidylinositol 3-kinase catalytic subunit type 3 (PIK3C3)/VPS34 and ATG14L. The post-translational modifications of Beclin1 and its complex are crucial for the control and regulation of autophagy [47]. Autophagy is modulated by mammalian targets of rapamycin (mTOR)-dependent and mTOR-independent signaling pathways. mTOR is a negative regulatory factor of autophagy, and the protein kinase B (PBK)/mTOR pathway and mitogen-activated protein kinase (MAPK)/mTOR pathway can suppress autophagy. In contrast, p53 and adenosine monophosphate-activated protein kinase (AMPK) inhibit mTOR, forming a positive autophagy pathway of AMPK/mTOR and p53/mTOR [48]. mTOR-independent autophagy pathways mainly include the ROS/c-Jun N-terminal kinase (JNK) oxidative stress pathway and the CCAAT enhancer-binding protein homologous protein (CHOP) pathway [49].

### 3.2. Autophagy in Mastitis

Pathogenic microorganisms can cause mastitis, which leads to inflammation at the site of infection and can also damage protective barriers such as BMECs [10]. Autophagy, which is an important part of the innate immune system, can directly mediate the removal of pathogens and alleviate inflammatory damage [50]. Studies have shown that autophagy can ameliorate inflammatory damage by maintaining the stability of organelles. For example, inhibition of autophagy can lead to abnormal mitochondrial aggregation in macrophages, resulting in increased ROS and NOD-like receptor protein 3 (NLRP3) inflammasomes, suggesting that autophagy may regulate the inflammatory response by maintaining mitochondrial homeostasis [51]. Autophagy negatively regulates inflammasome activation, which can reduce the levels of IL-1β and IL-18 and relieve inflammatory damage in the body [12]. Research by Sugimoto and others has shown that autophagy protects BMECs from invasion by *Streptococcus agalactiae* (*S. agalactiae*) [52]. Studies have shown that sodium valproate (VPA) can promote autophagy in BMECs by enhancing the level of LC3-II, reducing the level of P62, and alleviating the inflammatory response caused by γ-D-glutamyl-meso-diaminopimelic acid (iE-DAP) [53].

In addition to its protective role, autophagy can also be utilized by certain pathogens for proliferation or to evade host immune clearance, thereby enhancing their survival or pathogenicity and exacerbating host infection and damage. For example, *S. agalactiae* can activate autophagy through the PI3K/Akt/mTOR pathway, and the amount of intracellular pathogen is reduced after inhibiting autophagy with 3-methyladenine (3-MA), suggesting that autophagy is used to promote its survival in BMECs [54]. UFL1 is an important regulatory factor in NF-κB signal transduction and the cellular stress response. Li et al. found that the lack of UFL1 aggravated autophagy and the inflammatory response induced by LPS; in contrast, the overexpression of UFL1 significantly reduced the abnormal autophagy and inflammatory response induced by LPS, revealing that UFL1 regulated autophagy and alleviated the inflammatory response and damage in BMECs [55]. Wang et al. found that accumulated autophagosomes do not promote the clearance of *S. aureus*, suggesting that the formation of autophagosomes promotes the replication of *S. aureus* [56]. Schnaith et al. found that *S. aureus* expressing adhesion-related factors such as agr could inhibit the maturation of the autophagosome, which was conducive to the escape of *S. aureus* from the autophagosome to the cytoplasm, eventually causing the death of the host cell [57]. Maria et al. found that α-hemolysin secreted by *S. aureus* could induce autophagy, but autophagic vesicles induced by toxins could not fuse with lysosomes normally [58]. Chen et al. reported that *Streptococcus lutetiensis*, a new pathogen that causes bovine mastitis, induces autophagy in BMECs by increasing oxidative stress, which is the key to its pathogenesis [59]. Bovine macrophage infection with *S. aureus* induces mitochondrial autophagy through the PINK1/Parkin pathway, which is a mechanism used by bacteria to escape macrophage-induced death [60]. *S. aureus* induces mitochondrial autophagy in BMECs by activating the p38-PINK1-Parkin signaling pathway to promote their survival in cells [61].

### 3.3. Potential Therapeutic Applications of Autophagy in Mastitis

**Figure 3:**
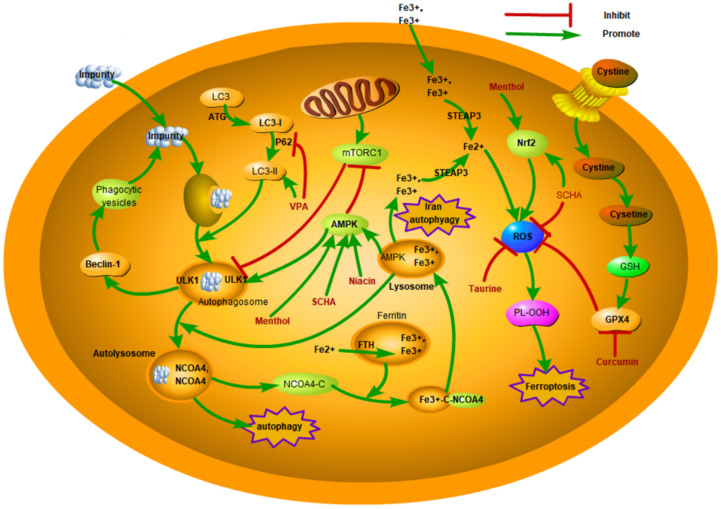
Therapeutic targets of core autophagy and ferroptosis proteins.

Given the important role of regulating autophagy in the pathogenesis of mastitis, autophagy is expected to be a therapeutic target for mastitis (Figure 3, Table 1). Santos et al. found that *Brassica oleracea* may inhibit dairy cow mastitis by interfering with the mechanism of action of the mTOR and TP53 genes [62]. Schisandrin A (SCHA) is a dibenzocyclooctadiene lignan extracted from Schisandra chinensis and has anti-inflammatory and antioxidative effects. Xu et al. found that SCHA inhibits the LPS-induced inflammatory response by triggering the NRF2 signaling pathway and induces autophagy by inhibiting the mTOR signaling pathway and triggering the AMPK-ULK1 signaling pathway, thus reducing the level of proinflammatory mediators and damage to the mammary gland and ultimately suppressing LPS-induced mastitis in mice. The results showed that SCHA could be used in mastitis treatment [30]. Guo et al. found that niacin could phosphorylate AMPK and trigger GPR109A, promote the nucleation and autophagy of NRF-2, and thus decrease the inflammatory response of BMECs induced by LPS [31]. In addition, niacin can enhance the blood–milk barrier and reduce mastitis by targeting GPR109A through AMPK/autophagy and AMPK/NRF2. GPR109A can markedly decrease macrophage supernatant-induced damage to BMECs [32]. These studies prove that niacin is of great value in treating mastitis. Zang et al. found that adequate selenium supplementation could increase the level of autophagy in RAW264.7 macrophages, inhibit the transcription of NF-κB and MAPK, and reduce the proliferation of *S. aureus* in RAW264.7 cells [33]. Wang et al. found that selenium promoted the fusion of lysosomes and autophagosomes, alleviated the obstruction of autophagic flux, and decreased the intracellular proliferation of *S. aureus*, which provides a foundation for the treatment and control of clinical *S. aureus* mastitis [50]. Menthol is a terpenoid compound with multiple bioactivities, and a large number of studies have shown that menthol has remarkable anti-inflammatory effects. Liu et al. found that menthol promoted autophagy by triggering the AMPK/ULK1 and AMPK/NRF-2 pathways, thereby inhibiting the level of inflammatory factors and proinflammatory enzymes and significantly reducing the inflammatory response of BMECs induced by LPS. This makes menthol a potential drug for mastitis [34]. Studies have shown that taurine suppresses mTOR signaling by triggering PTEN, thus causing autophagy, accelerating the degradation of intracellular *S. uberis*, reducing intracellular bacterial load, inhibiting overactivation of the inflammatory response, and alleviating the damage caused by *S. uberis* in BMECs [12].

## 4. Pyroptosis

Pyroptosis is a kind of RCD caused by infectious or noninfectious factors that is closely related to inflammation and is regulated by genes. Pyroptosis was discovered by Zychlinsky et al. in 1992, who observed macrophage lysis caused by Shigella infection, which was defined as PCD mediated by the gasdermin family by Shao Feng’s team [63,64]. Pyroptosis is a proinflammatory RCD mode whose occurrence and development depend on caspases. It is characterized by gasdermin D (GSDMD)-mediated cell membrane pore formation, cell swelling, and rapid lysis, followed by the release of a large number of inflammatory mediators, such as IL-1β and IL-18, thus initiating a cascade of expanded inflammatory responses [65].

### 4.1. Overview of the Core Mechanisms of Pyroptosis

The classical pathway mediated by caspase-1 and the nonclassical pathway mediated by caspase-4/5/11 constitute the main pathways for the occurrence of pyroptosis. In the classical pathway, intracellular inflammasomes, including NLRP3, AIM2, and pyrin, are activated by exogenous pathogens and endogenous danger signals. Activated inflammasomes activate caspase-1 by binding pro-caspase-1 to downstream apoptosis-associated spot-like proteins containing caspase-1 activation and recruitment domains, forming a multiprotein complex. Activated caspase-1 cleaves GSDMD to produce the N-terminus, which forms small pores in the cell membrane that promote the release of cytoplasmic contents such as IL-1β and IL-18, thereby inducing inflammatory effects. In the nonclassical pathway, lipopolysaccharides, which are a component of the cell wall of gram-negative bacteria, bind directly to macrophage caspase-4/5/11. Activated caspase-4/5/11 lyses GSDMD, directly triggering pyroptosis, and activated NLRP3 inflammasomes induce the release of IL-1β and IL-18 [66]. Wang et al. found that activated caspase-3 could also specifically cleave GSDME, causing GSDME to generate an N-terminus and C-terminus, mediating pyroptosis [67]. Kambara et al. found that in aging neutrophils, neutrophil elastase (ELANE) is released from cytosolic granules into the cytoplasm. ELANE-mediated GSDMD cleavage is located upstream of the caspase cleavage site and produces fully active ELANE-derived N-fragments, which induce cell death in the same manner as GSDMD and mediate pyroptosis in neutrophils [68].

### 4.2. Pyroptosis in Mastitis

The pathogenesis of mastitis is accompanied by inflammation and cell damage [69]. BMECs have immune activity and can activate NLRP3 inflammatory mediators during mastitis, and then the activated NLRP3 inflammasome causes pyroptosis, thereby releasing proinflammatory cytokines and further expanding the inflammatory response. Wei et al. found that neutrophil extracellular traps (NETs) and their histones have obvious cytotoxic effects on BMECs, and histones significantly increase the activation of caspase1, caspase3, and NLRP3, leading to pyroptosis and cell damage [70]. The effect of pyroptosis on mastitis has two sides. On the one hand, when pathogenic microorganisms invade the body and cause mastitis, the immune protection mechanism in the mammary gland is activated, and the inflammasome is activated to induce pyroptosis, thus promoting the inflammatory response to eliminate pathogenic microorganisms. On the other hand, when pyroptosis is excessive or out of control, it causes a serious systemic inflammatory response and may lead to multiorgan failure [13]. Pathogenic microorganisms also use pyroptosis to evade the immune system; for example, *S. uberis* infection causes tissue damage by inducing pyroptosis [24]. Wang et al. found that *S. aureus* activates NLRP3 through the K^+^ efflux pathway and causes MAC-T-cell pyroptosis [71].

### 4.3. Potential Therapeutic Applications of Pyroptosis in Mastitis

Dioscin is a natural steroidal saponin that has anti-inflammatory effects. Xin et al. found that dioscin reduced pyroptosis by triggering AMPK/NRF2 and suppressing the NF-κB signal transduction pathway, significantly alleviating LPS-induced mastitis in mice [35]. In *E. coli* infection, Lactobacillus rhamnose-LGR-1 inhibited the self-hydrolytic activation of caspase-1 and decreased the expression of IL-18 and IL-1β by inhibiting the activity of the NLRP4 and NLRC4 inflammasome during ASC dependence, thus inhibiting the inflammatory damage of BMECs caused by *E. coli*. In addition, *Lactobacillus rhamnosus* GR-1 also inhibited *E. coli*-induced pyroptosis, in part by attenuating NLRC4 and nonclassical Caspase-4 activation independent of ASC [36]. UFL1, an important regulator of cellular stress, can inhibit the activation of the NLRP3 inflammasome by regulating NF-κB signaling and ROS production and reduce LPS-induced pyroptosis, thus having anti-inflammatory and protective effects on BMECs (Figure 2, Table 1) [72].

## 5. Ferroptosis

Ferroptosis is a unique iron-dependent RCD that is associated with BMEC dysfunction, unlike apoptosis, necroptosis, and pyroptosis [37,73]. Studies have shown that high levels of ROS in cells are a direct cause of ferroptosis [74]. During ferroptosis, mitochondria are shrunken, membrane density increases, and iron ions accumulate excessively in cells, which leads to increased ROS, decreased GSH levels, and lipid peroxidation. At present, studies on ferroptosis mainly focus on tumor inhibition and antiviral immune response [75], and there have been few reports on the relationship between ferroptosis and mastitis in dairy cows.

### 5.1. Overview of the Core Mechanisms of Ferroptosis

Ferroptosis is considered to be a pathological process closely related to metabolism and is mainly regulated by iron metabolism, amino acid metabolism, and lipid metabolism, which jointly participate in the regulation of ferroptosis, mediate the occurrence of ferroptosis, and ultimately execute cell death through iron-dependent intracellular lipid peroxide accumulation [76]. The cyst(e)ine/GSH/GPX4 system is thought to be involved in the classic pathway of ferroptosis [77]. Fe^2+^ catalyzes the peroxidation of polyunsaturated fatty acids (PUFAs), the Fenton reaction, and the production of ROS. Therefore, the accumulation of Fe^2+^ is one of the most important driving factors of ferroptosis. Since the concentration of Fe^2+^ is regulated by iron metabolism, any factor that interferes with iron metabolism may affect the occurrence of ferroptosis [78]. Cell membranes and organelle membranes are highly susceptible to ROS damage due to their rich PUFAs. Lipid peroxidation is a necessary part of ferroptosis [79]. In recent years, several nonclassical pathways have also been reported to participate in the regulation of ferroptosis, including the mevalonate pathway, the GCH1/BH4/DHFR system, the TXN system, and the peroxisome [80]. In addition, ferroptosis is regulated by multiple transcription factors and epigenetic regulatory molecules.

### 5.2. Ferroptosis in Mastitis

Mastitis inflammation is accompanied by the production of arachidonic acid, whose metabolites are essential for cyclooxygenase (COX) and lipoxygenase (LOX) and whose activity leads to the formation of proinflammatory substances such as prostaglandins, thromboxins, and leukotrienes [81]. The presence of inflammatory mediators produced by arachidonic acid metabolism in ferroptosis is very similar to that in mastitis. In a recent study, Zhang et al. found that LPS could significantly upregulate the levels of ferritin heavy chain 1 (FTH1) and heme oxygenase-1 (HMOX1) and iron accumulation in MAC-T cells in vitro, which is similar to the effect of erastin [82]. Subsequently, proteomics was used to identify 302 differentially expressed proteins and 11 GO terms associated with iron homeostasis in dairy cow mastitis mammary tissue. The expression of HMOX1 and FTH1, two key molecules in the ferroptosis signaling pathway, was significantly increased, indicating that ferroptosis was involved in the pathogenesis of mastitis [82]. Another transcriptome study of peripheral blood leukocytes suggested that alternative splicing of ferroptosis-related genes affected the development of subclinical mastitis [83]. Using a mouse mastitis model and mammary epithelial cells (MMECs), Bao et al. found that *S. aureus* induces ferroptosis in MMECs in a dose-dependent manner, which is closely related to ROS production, ER stress, and autophagy activation and serves as a trigger of ferroptosis [73].

### 5.3. Potential Therapeutic Applications of Ferroptosis in Mastitis

Curcumin has been safely and broadly used as a natural food coloring for a long time, and many studies have shown that it has anticancer, antioxidant, and anti-inflammatory properties. Li et al. found that different doses of curcumin induced different antioxidant effects in different cells. After treatment with 10 µM curcumin for 24 h, MAC-T cells were protected from LPS-induced oxidative stress by activating the NFE2L2 pathway and alleviating the cellular inflammatory response [37]. In the cell lines MAC-T and MCF-7, curcumin can induce oxidative stress-induced ferroptosis by upregulating HMOX1 and downregulating GPX4 expression (Figure 3, Table 1) [37]. Schisandrin B (Sch B) is the most active ingredient in Schisandrae Chinensis Fructus and has been proven to have the ability to inhibit inflammation and bacteria. SCB can resist *S. aureus*-induced ferroptosis via up-regulating the SIRT1/p53/SLC7A11 signaling pathway, which subsequently inhibits inflammation-associated cytokines and alleviates mastitis in the mammary gland tissues [38].

## 6. Necroptosis

Traditionally, apoptosis was thought to be regulated, whereas necrosis was unregulated and difficult to reverse. However, recent studies have shown that necroptosis is a mechanism of cell necrosis that occurs when apoptosis is inhibited. Necroptosis is characterized by cell swelling, membrane lysis, and the release of cell contents, and damage-associated molecular patterns (DAMPs) cause acute local tissue inflammation [15].

### 6.1. Overview of the Core Mechanisms of Necroptosis

The regulatory network of necroptosis is very complex and is related to other forms of death. The molecular pathways of necroptosis include initiation, transmission, and execution, which eventually lead to cell death. Necroptosis is mediated by a number of death receptors, including CD95 (or FAS), TNF receptor 1 (TNFR1), TNFR2, and TNF-related apoptosis-inducing ligand receptor (TRAIL) 1 and 2 [84]. Currently, the most exhaustive research on the regulation of necroptosis is the signaling pathway mediated by TNF and TNFR1 [85]. TNF-α binds to TNFR1, resulting in the trimerization of its receptors, and then a series of proteins, such as TNF receptor-related death domain (TRADD), RIP1, TNF receptor-associated factor 2, intracellular apoptosis inhibitor protein 1 (clAP1), and cIAP2, are recruited to TNFR1 to form complexes [86]. When RIPl binds to Caspase 8, it leads to cell apoptosis. However, when caspase 8 is absent or inhibited, RIPl and RIP3 can interact to phosphorylate and form necrosomes, which transition from the cytoplasm to the plasma membrane and organelle membrane and form permeable pores on the membrane structure, destroying the integrity of the membrane. This leads to the release of cell contents as well as the influx of calcium and sodium ions, which eventually induce necroptosis [87,88].

### 6.2. Necroptosis in Mastitis

Necroptosis has been shown to exist in many diseases in recent years and can cause local inflammation. HE staining of healthy and clinical mastitis breast tissue showed that in the breast tissue in the mastitis area, the acinar compartment had lost its original shape, the vesicles were significantly atrophied and interspersed with detached epithelial tissue, and there was significant hyperplasia of interstitial cells [15]. By detecting inflammatory cytokines (TNF-α, IL-1β, IL-6) and necroptosis-related factor genes (caspase8, RIPK1, and RIPK3), mRNA expression, and MLKL and *p*-MLKL protein expression and differential analysis, it was found that there was necroptosis in the mammary tissue of dairy cows with mastitis. In addition, the knockdown of MLKL in an in vitro necroptosis inflammatory cell model with a mixture of TNF-α, cyclohexanone, and fluoromethyl ketone reduced the sensitivity of BMECs to necroptosis, confirming the in vivo findings [15]. Yin et al. found that *Klebsiella* infection increased the colocalization and interaction of RIPK3 and RIPK1, activated RIPK3, and upregulated the phosphorylation level of MLKL, thus triggering necroptosis in EpH4-Ev cells [39]. LncRNAs may also be involved in regulating necroptosis in BMECs [89].

### 6.3. Potential Therapeutic Applications of Necroptosis in Mastitis

In recent years, flavonoids and their analogs have been used as feed additives to promote the growth and development of dairy cows due to their good antibacterial and antioxidant effects. Bamboo leaf extract is rich in flavonoids, among which a large number of natural polyphenols exist in the form of free aglycones or glycoside conjugates. Wei et al. examined the microbiome and metabolome of milk and found that adding antioxidants of bamboo leaf (AOB) could increase the protein content in milk, reduce the somatic cell count (SCC), change the structure of the bacterial community in milk, and enrich the necroptosis signaling pathway [90]. Yin et al. reported that taurine could suppress RIPK1 and significantly relieve inflammation, injury, and necroptosis caused by *Klebsiella* infection, suggesting that necroptosis may be a therapeutic target for alleviating inflammation and injury caused by *Klebsiella* infection (Figure 2, Table 1) [39].

## 7. Conclusions

The withering of flowers and the falling of branches are not only the end of life but also for redder flowers and greener leaves in the coming year. The same is true for the death of cells. It is through cell death that the organism regulates normal functions, maintaining intracellular homeostasis. Cell death is also strongly related to the occurrence of diseases. Disordered regulatory mechanisms of cell death can lead to the occurrence of clinically relevant diseases, including inflammatory diseases, neurodegenerative diseases, and cancer. Although the transduction pathways between apoptosis, autophagy, necroptosis, pyroptosis, and ferroptosis are different, a series of studies have shown that there is crosstalk among them. For example, cells stimulated by TNF and oxidative factors can promote the release of apoptotic factors from mitochondria, thereby inducing cell apoptosis, but inhibiting apoptosis can also cause autophagy. Some damaged mitochondria in apoptotic cells can be eliminated by autophagy, thereby reducing apoptosis. However, caspase-8 inhibition can lead to necroptosis [91]. If the GSDME protein is present in the cell at this time, it will quickly cause the cell to transition from apoptosis to pyroptosis [65]. Similarly, autophagy can inhibit pyroptosis by reducing the production of inflammasomes, and the autophagy-initiating kinase ULK1 is involved in controlling RIP1-mediated necroptosis [92]. Apoptosis can also be transformed into ferroptosis, which can increase the sensitivity of cells to apoptosis. Studies on the correlation between autophagy and ferroptosis have shown that oxidative stress damage associated with ferroptosis can induce lysosome rupture, phagosomes formed during autophagic death bind to lysosomes and are degraded, and the Kelch-like epichlorohydrin-related protein 1 (Keap1)/Nrf2 signaling pathway may be a connection between ferroptosis and autophagy [93]. Recently, Malireddi et al. proposed a comprehensive programmed cell death mechanism called pan apoptosis, which has also been reported in mastitis [94,95]. Therefore, it is thought that there is a relationship between mutual restriction and dynamic balance among apoptosis, autophagy, programmed necroptosis, pyroptosis, and ferroptosis.

Mastitis is one of the most important and expensive diseases in the dairy industry. An increasing number of studies have been conducted to prevent and treat clinical mastitis from distinct perspectives, such as the development of new drugs, therapies, and vaccines [96]. However, mastitis remains a difficult issue that plagues the dairy industry worldwide. New compounds or antibiotics are urgently needed due to the high prevalence of mastitis and the subsequent overuse of antibiotics. There is increasing evidence that the destruction of homeostasis caused by pathogen infection and immune-mediated disorders, as well as mineral deficiency, are key causes of bovine mastitis [82]. Coincidentally, iron competition between microorganisms and host cells is the basis for the development of infection and inflammation [97]. For example, ferroptosis can be recognized by immune cells, triggering a series of specific or inflammatory reactions [98]. In addition, in mastitis, inflammation is accompanied by phospholipase activation, and phospholipase produces arachidonic acid through membrane phospholipids [81]. Arachidonic acid metabolites are necessary for COX and LOX enzyme systems. The metabolism of arachidonic acid into bioactive proinflammatory mediator precursors through three metabolic pathways is a key inflammatory pathway [81]. These characteristics of mastitis are highly consistent with the characteristics of ferroptosis. To fully utilize the therapeutic potential of RCD during mastitis, it is necessary to further study the common connections between different cell death pathways and target their key factors, which is expected to achieve a breakthrough in the treatment of mastitis. Similarly, in the future, it will be necessary to study the respective proportion of various forms of cell death during mastitis and to clarify the contribution of various forms of cell death to mastitis. Since cell death during mastitis is a multifactorial and multilink signal transduction process, it is necessary to further understand the activation conditions and relationships of signal transduction pathways mediated by various factors in various forms of cell death to provide a new direction for the prevention and treatment of mastitis.

## Figures and Tables

**Table 1:** Application of regulated cell death in mastitis.

RCD Item	Drugs	Immune Response	Animal Model/Cell Lines	Functional Mechanism	Clinical Application Value	References
Apoptosis	Gambogic acid (GA)	Not clear	Primary and HC11 murine mammary epithelial cells	GA inhibits apoptosis via Bcl-2, caspase-3, and 9.	GA has potential benefits as a potential treatment for mastitis.	[25]
	Matrine	Matrine has immune-enhancing effects	BMEC cell line (MAC-T)	Matrine downregulated the levels of cleaved caspase-3, cleaved caspase-8, and cleaved caspase-9.	Matrine has potential value against cow mastitis caused by the toxin PVL.	[20]
	Baicalin	Baicalin has immune-enhancing effects	BMEC cell line (MAC-T)	Baicalin downregulated the expression of cleaved caspase-9.	Baicalin has potential value against cow mastitis caused by the toxin PVL.	[20]
	Tea tree oil (TTO)	Not clear	Isolated bovine mammary epithelial cells	TTO inhibits the production of caspase-3.	TTO has a protective effect against LPS-induced mastitis.	[26]
	MSC-ACE2	MSC-ACE2 has immune regulatory activity	EpH4-Ev cells (Mouse mammary epithelial cells)	MSC-ACE2 reversed the LPS-induced down-regulation of Bcl2 and the up-regulation of Bax and caspase-3	MSCs overexpressing ACE2 are expected to serve as a potential strategy for mastitis treatment.	[21,27]
	Sodium butyrate (SB)	Not clear	BMEC cell line (MAC-T)	SB reduced LPS-induced apoptosis by inhibiting the NF-kB and caspase/Bax signaling pathways.	SB may be used as a therapeutic agent for mastitis.	[28]
	Procyanidin B2 (PB2)	Not clear	BMEC cell line (MAC-T)	PB2 increased expressions of Bax, Bcl-2, Bax/Bcl-2, and Cyto-c while decreasing expressions of cleaved caspase-3.	PB2 is an antioxidant to improve HS-induced mitochondrial dysfunction and inflammation in BMEC.	[29]
Autophagy	Schisandrin A (Sch A)	Not clear	C57BL/6 mice and mouse mammary epithelial cells (mMECs)	Sch A induces autophagy by suppressing the mTOR signaling pathway and activating the AMPK-ULK1 signaling pathway.	Sch A is promising for use in the treatment of mastitis.	[30]
	Niacin	Niacin increases innate immunity	Lactating dairy cows and primary bovine mammary epithelial cells (BMECs)	Niacin might promote autophagy by activating the GPR109A/AMPK/NRF-2 signaling pathway.	Niacin alleviates mastitis in dairy cows.	[31,32]
	Selenium	Selenium regulates immunity	RAW264.7 macrophages	Selenium improves autophagy by modulating the expression of LC3 II and p62.	Not clear	[33]
	Menthol	Not clear	Primary bovine mammary gland epithelial cells (BMECs)	Mnthol activates the AMPK-ULK1 pathway to initiate the onset of autophagy and maintains the level of autophagy through the AMPK-Nrf-2 pathway.	Not clear	[34]
	Taurine	Taurine regulates innate immunity	BMEC cell line (MAC-T)	Taurine activates autophagy in an mTOR-dependent manner.	It provides theoretical support for the development of prophylactic strategies for pathogen	[12]
Pyroptosis	Dioscin	Not clear	BALB/c mice	Dioscin reduced pyroptosis by triggering AMPK/NRF2 and suppressing the NF-κB signal pathway.	It provides a new potential therapy of dioscin for the treatment and prevention of mastitis.	[35]
	*Lactobacillus rhamnosus* GR-1	*L. rhamnosus* GR-1regulates the immune response	BMEC cell line (MAC-T)	*L. rhamnosus* GR-1 suppresses *E. coli*-induced pyroptosis through attenuation of NLRC4 inflammasome and non-canonical caspase-4 activation, independent of ASC.	*L. rhamnosus* GR-1 represents a potentially promising therapeutic agent in *E. coli*-associated mastitis.	[36]
Ferroptosis	Curcumin	Not clear	BMEC cell line (MAC-T)	Curcumin induces ferroptosis by upregulating HMOX1 and downregulating GPX4 expression.	Curcumin has potential benefits as a potential treatment for mastitis.	[37]
	Schisandrin B (SCB)	Not clear	Balb/c mice	SCB attenuates *S. aureus*-induced ferroptosis via up-regulating SIRT1/p53/SLC7A11 signaling pathway.	SCB shows great potential to resist inflammation in *S. aureus*-induced mastitis.	[38]
Necroptosis	Taurine	Taurine regulates the innate immune	EpH4-Ev cells (mouse mammary epithelial cells)	Taurine could suppress the RIPK1/RIPK3/MLKL signaling pathway and subsequently relieve necroptosis caused by Klebsiella infection.	It provides a basis for using Taurine to prevent Klebsiella infection and the development of novel prophylactic strategies.	[39]

## Abbreviations

The following abbreviations are used in this manuscript:

RCD	regulated cell death
PCD	programmed cell death
ROS	reactive oxygen species
LPS	lipopolysaccharide
BMECs	bovine mammary epithelial cells
GMECs	goat mammary epithelial cells
Cytc	cytochrome C
TNF	tumor necrosis factor
PVL	Panton–Valentine leukocidin
MAPK4	mitogen-activated protein kinase 4
MSCs	mesenchymal stem cells
ACE2	Angiotensin-converting enzyme 2
ATGs	autophagy-associated genes
CMA	chaperone-mediated autophagy
AMPK	adenosine monophosphate-activated protein kinase
NLRP3	NOD-like receptor protein 3
NETs	neutrophil extracellular traps
PUFAs	polyunsaturated fatty acids
FTH1	ferritin heavy chain 1
HMOX1	heme oxygenase-1
DAMPs	damage-associated molecular patterns
TRAIL	TNF-related apoptosis-inducing ligand receptor
TRADD	TNF receptor-related death domain
Keap1	Kelch-like epichlorohydrin-related protein 1
SCC	somatic cell count
AOB	antioxidants of bamboo leaf
MMP	mitochondrial membrane potential
PIK3C3	phosphatidylinositol 3-kinase catalytic subunit type 3
